# Rehabilitation therapy versus drug-only therapy in patients with multiple sclerosis

**DOI:** 10.55730/1300-0144.5776

**Published:** 2023-07-17

**Authors:** Florin Mihai MARCU, Doriana CIOBANU, Ioan Cosmin BOCA, Elena SIRBU, Paul Alexandru DEME, Nicolae Catalin HRENIUC, Dorina IANC

**Affiliations:** 1 Department of Psychoneuroscience and Rehabilitation, Faculty of Medicine and Pharmacy, University of Oradea, Oradea, Romania; 2 Human Performance Research Center, University of Oradea, Oradea, Romania; 3 Department of Physical Therapy and Special Motricity, Faculty of Physical Education and Sport, West University of Timişoara, Timişoara, Romania; 4 Department of Orthopedics and Traumatology, Faculty of Medicine, “Vasile Goldiş” Western University of Arad, Arad, Romania; 5 Department of Neurology, Faculty of Medicine, “Vasile Goldiş” Western University of Arad, Arad, Romania

**Keywords:** Multiple sclerosis, physical therapy, rehabilitation treatment, occupational therapy

## Abstract

**Background/aim:**

The treatment for multiple sclerosis (MS) does not cure the disease, but it is intended to reduce the intensity, duration, and frequency of symptoms. Rehabilitation therapy (RT), including an individualized physical therapy program (PTP) and adapted occupational therapy (OT), has benefits in terms of aerobic capacity, muscle strength, coordination, and ability to perform activities of daily living (ADL). The primary objective of this study was to examine the efficacy of RT comprising PTP, OT, and drug treatment (DT) versus DT alone in patients with MS. Another objective was to highlight the importance of continuing the PT and OT at home, in the long term, practically for their entire life.

**Materials and methods:**

Between 2020 and 2022, a follow-up observational study was conducted that included 77 patients diagnosed with MS, independent in terms of ability to perform ADL, divided into two groups: group A (39 patients who complied with the RT) and group B (38 patients who did not comply). At the beginning and end of the study, the following parameters were assessed: timed walk for 25 feet [Timed 25-Foot Walk test (T25FW test)], dexterity of the upper limbs [9-Hole Peg Test (9HPT)], and cognitive function [Paced Auditory Serial Addition Test (PASAT)].

**Results:**

Significant improvement in the experimental group was observed regarding the mobility and the performance of leg function (T25FW, p < 0.05) and finger dexterity (9HPT, p < 0.05) for the dominant hand.

**Conclusion:**

The current study proves the importance of combining DT with RT in MS therapy with clear benefits in regaining muscle strength in the lower limbs, thus improving coordination and balance while walking and improving dexterity in the dominant hand.

## 1. Introduction

Also known as “the disease of a thousand faces”, multiple sclerosis (MS) can take different forms: new symptoms appear either in sporadic attacks, relapsing forms or worsening over time, progressive forms [1]. People living with MS also experience “invisible’ symptoms,” which include, but are not limited to, fatigue, mood changes, cognitive changes, physical and emotional pain, spasticity, bowel/bladder dysfunction, sexual dysfunction, and vision changes [2]. There is no etiological therapy known to treat the cause of this disease.

The drug treatment (DT) of MS is mainly symptomatic and aims to improve the patient’s quality of life (QoL) by reducing the intensity, duration, and frequency of clinical manifestations [3]. The DT includes 3 types of drugs: corticosteroids, drugs that help to reduce inflammation and delay the damage of myelin sheaths; adjuvant drugs to relieve symptoms; and immunomodulatory and immunosuppressive preparations for reducing the autoimmune processes of myelin destruction and postponing the appearance of motor deficiencies. These are disease modifiers, which reduce the incidence of the disease and delay its progression in some patients [4]. The most frequently used symptomatic drugs are antidepressants, analgesics, and preparations for improving intestinal motility [5].

Rehabilitation therapy (RT), which is mainly a combination of adapted occupational therapy (OT) and a specific physical therapy program (PTP), increases the level of functionality and independence, and the patients’ QoL [6]. In patients with MS, the PTP has a favorable effect on muscle strength, fatigue, joint mobility, spasticity, body weight, and mood. Moreover, it can have a preventive effect in the onset of bedsores, constipation, and complications related to osteoporosis. Depending on the degree of the disability, an aerobic PTP slows down the progression of the disease and reduces the symptoms [7,8].

The primary objective of the present study was to emphasize the efficacy of RT comprising a PTP, OT, and DT versus DT alone in patients with MS. The other objective was to highlight the importance of continuing the PT and OT at home, in the long term, practically for their entire life.

## 2. Materials and methods

### 2.1. Study design and participants

Seventy-seven patients were involved in the study. They were admitted at the Emergency Clinical County Hospital of Oradea, Romania, and they gave their written consent to take part in the study. These patients were selected based on subjective and objective anamnesis criteria. The study was conducted over more than 2 years, between June 2020 and July 2022. Initially, there were 80 participants, but 3 were excluded during the research for various reasons.

The inclusion criteria for this clinical study were as follows: confirmed MS diagnosis, written consent of the patients to take part, compliance with DT and RT throughout the study, compliance with the assessment of the monitored parameters, and moderate neurological disability, according to the Expanded Disability Status Scale (EDSS), with a score up to 4.5. The exclusion criteria were refusal to participate, alteration of the patient’s general condition during the study regardless of the cause of this issue (inability to perform the weekly PTP), any type of complications, neoplasm, debilitating comorbidities, illness, and the presence of a disability significant enough to affect the performance of activities of daily living (ADL). Those whose medication prevented them from performing the PTP 3 times a week were also excluded during the study. Specifically, there were two patients who took sedatives to treat anxiety (Rivotril, active ingredient clonazepam) and antidepressants to treat depression (Trittico, active ingredient trazodone).

The EDSS is used to quantify disability in MS and monitor changes in disability levels over time, being widely used in the assessment of people with MS [9,10]. The Multiple Sclerosis Functional Composite (MSFC) score is one of the most used, standardized and quantitative, multidimensional assessment tools for MS [11]. The three component steps of the MSFC are a timed walk for 25 feet (T25FW test), the 9-Hole Peg Test (9-HPT), and the Paced Auditory Serial Addition Test (PASAT) [12].

All participants in the study received DT, initiated from the start of the study. The RT consisted of a PTP performed initially in the hospital for 8 weeks with three weekly sessions and OT adapted activities. After this period, the patients were advised to continue the PTP and OT at home. For subjective reasons of medical discipline (availability, adherence, health costs, etc.), the indication to undergo RT at home was not followed by all patients. Depending on this factor, they were divided into two groups: group A – study group (39 patients treated with DT and RT), and group B – control group (38 patients treated with DT only).

The assignment to the two groups was done according to each individual patient. Willingness to participate and ability to comply with the PTP schedule and indications for ADL, as well as financial willingness to make adaptive changes at home were considered. To exclude patient selection bias, we considered the potential for natural recovery from MS and we excluded patients with adjuvant therapies that have a major impact [13]. Thus, only patients with MS for more than 6 months were included because the potential for natural recovery decreases with illness duration and eventually approaches zero.

### 2.2. Assessments

In order to highlight the positive effect of RT, the evolution of the following parameters was monitored:

1. The T25FW test, a quantitative function test to assess lower limb mobility. The score for this test is the mean of two attempts. Depending on the severity of the disability, patients may use assistive devices when doing this task; the assessments were performed by the same physiotherapist [14,15].
2. The 9HPT is a simple test of fine motor coordination of the upper limbs, finger dexterity. The score for each hand is the mean of two trials. Two consecutive trials with the dominant hand are immediately followed by two consecutive trials with the nondominant hand; the assessments were performed by the same physiotherapist [16,17].
3. The PASAT is used on a large scale for assessing the cognitive performance of patients with MS. The score is the total number correct out of 60 possible answers; the assessments were performed by the same physiotherapist [17,18].

The assessments of the parameters monitored were performed in the morning at around 10 a.m., before starting the DT, at time T0 and after 1 year, respectively, when the study ended, at time T1.

### 2.3. Interventions

Each mpatient in group A followed a treatment between DT and RT for 1 year. The DT, adjusted depending on the stage of the disease and the patient’s compliance, consisted of treatments:

- that modify the evolution of MS: immunomodulatory drugs: Copaxone, active ingredient: glatiramer acetate;
- that prevent exacerbation: i.v. glucocorticoids: Solumedrol, active ingredient: methylprednisolone sodium succinate;
- that are symptomatic and rehabilitative: adjusted and individualized as needed, for example: vitamins, NSAIDs, antidepressants, and sedatives.

The RT consisted of a PTP performed initially in the hospital for 8 weeks with 3 weekly sessions and OT adapted activities. After leaving hospital, the patients continued to perform at home 3 weekly sessions, which were monitored by physiotherapists from the Multiple Sclerosis Foundation in the city and student volunteers. All exercises in the PTP were aerobic to avoid and delay the onset of fatigue. No work was performed when pain was felt and the pain limit was not exceeded during joint mobilizations. In terms of gradualness, we worked from simple to complex, from easy to hard, gradually increasing the intensity of the exercises and the number of repetitions. The breaks between exercises to avoid fatigue were extremely important. The dosage and intensity of the exercise were set and progressed over time. Modification of the intervention was individually tailored.

The PTP was structured as follows: warm up (5 to 7 min), exercise program (30 to 35 min), and cool down (5 min). Each PTP session lasted 40 to 47 minutes and was performed 3 times per week. All exercises were adapted to the level of disability and individual characteristics of each patient. They were always reminded to keep their breathing flowing, both during the warm-up period and during the exercise period.

The exercise program involved the following objectives:

- *reducing spasticity* (7 min): hold–relax proprioceptive neuromuscular facilitation (PNF) technique, strengthening opposing muscle groups to inhibit the spastic muscles;
- *combating weakness* (7 min): resistance training exercises (elastic bands, weights, stationary bike, stair steppers), repeated contraction PNF technique for maintaining muscle strength in the legs, especially for anterior tibialis, quadriceps, gluteus medius and for strengthening synergistic muscles;
- *increasing balance* (7 min): rhythmic stabilization PNF technique, traditional balance exercises, core strengthening exercises, elements from Tai Chi (a martial art); progressively challenging manipulation of objects during standing and walking;
- *improving gait* (7 min): various types of walking, with changes in speed, step length, walking over obstacles, body-weight-supported treadmill training;
- *improving upper limb coordination* (7 min): progressively challenging manipulation of objects during sitting, elements from Tai Chi.

After a 10-min break, the patients moved on to the OT program, which lasted 20 min and focused on exercise for increasing independence, safety, and QoL during daily activities.

No single occupational therapy plan was used, and each person’s exercise regimen varied. The treatment plans changed over time, depending on how the person’s priorities changed and how the disease progressed.

The study was conducted according to the guidelines of the Declaration of Helsinki and it was approved by the Ethical Commission and the Ethical Council of the Emergency Clinical County Hospital of Oradea, Romania (registration no. 16479/02.06.2020, 16486/03.06.2020).

### 2.4. Study size

In order to establish the sample size, we used as primary reference the number of patients with MS who presented to the foundation where the study was carried out, as being the population size. We are referring to a number of 100. We also considered the 95% confidence interval, and a 5% margin of error and an assumed population ratio of 0.5. The z score for a 95% confidence interval is 1.96. In order to obtain the sample size according to these parameters, we used an online sample size calculator. The sample size for the chosen population size is 80.

### 2.5. Statistical analyses

For data analysis, we used the Statistical Package for the Social Sciences (SPSS) Evaluation version 15.0, issued by IBM SPSS Statistic, Oradea, Romania. For quantitative analysis of the numerical variables, we used mean and standard deviation, and for the categorical variables we used percentage and mean. We analyzed the normality of the data distribution using the Kolmogorov–Smirnov test. For the intergroup analysis of the initial values, we used the t-test for independent samples, as we had a normal data distribution (the Kolmogorov–Smirnov test, p ≥ 0.05). The chi-squared test for homogeneity was conducted in order to explore whether frequency counts were distributed identically across the two groups of patients, in terms of sex and environment.

In order to test if there was a significant difference between the two groups for the initial and final results, we used one-way ANOVA between the patients, as we had a normal data distribution (the Kolmogorov–Smirnov test, p ≥ 0.05). For the pretest–posttest analysis of the two groups we used one-way ANOVA with repeated measurements. In order to measure the effect size measure for both one-way ANOVA between patients and one-way ANOVA with repeated measurements, partial eta squared was used. In total, 95% confidence intervals were reported accordingly.

## 3. Results

The distribution of the patients’ parameters in each group was homogeneous (differences were not statistically significant) according to age (Table 1) (Kolmogorov–Smirnov test, p ≥ 0.05). Furthermore, there was no significant difference between the experimental and control groups regarding data homogeneity according to sex [x^2^ (1) = 0.027; p ≥ 0.05] or environment [x^2^ (2) = 0.105; p ≥ 0.05].

There was no significant difference between the groups regarding the initial values for the parameters monitored in the study, namely T25FW, 9HPT, and PASAT (Table 2).

The comparison of the final assessment results between the groups shows that there are significant differences regarding T25FW [F (1.75) = 10.440, p < 0.05] and 9HPT for the dominant hand [F (1.75) = 30.475, p < 0.05]. After 1 year, group A had improved more than group B with regard to mobility and performance of leg function (T25FW) and finger dexterity (9HPT) for the dominant hand (p < 0.05).

The analysis within group B showed significant differences between the pretest and posttest values for 9HPT for the nondominant hand [F (1.37) = −4.324, p < 0.05].

Within group A, there were significant differences between the pretest and posttest values for T25FW [F (1.38) = 16.165, p < 0.05], 9HPT for the dominant hand [F (1.38) = 13.726, p < 0.05], and PASAT [F (1.38) = −3.575, p < 0.05].

Patients in the experimental group experienced significant improvements in the following studied variables: mobility and performance of leg function, finger dexterity for the dominant hand, and cognitive performance (Table 3). The data presented in bold show a statistically significant difference between the groups (interaction) and within the groups (group A changes and group B changes).

## 4. Discussion

The overall objective of the present study was to determine whether PTP applied together with DT has a better effect than DT alone in patients with MS. The comparison between the groups in the study showed that the mobility and performance of leg function (T25FW) and finger dexterity (9HPT) for the dominant hand improved in subjects who continued with rehabilitation (SWCWR). The findings revealed no statistically significant difference between the two groups in finger dexterity for the nondominant hand (9HPT) or cognitive performance (PASAT).

Significant improvements were recorded in the mobility and performance of leg function, finger dexterity for the dominant hand, and cognitive performance in the SWCWR. Our results regarding the improvement of the function of the lower limb are consistent with those mentioned in the specialized literature [19,20], which show a positive effect of the PTP on stability, muscle strength, and walking in patients with MS.

As in other specialized studies, bicycle ergometry, arm–leg ergometry, and walking on a treadmill were performed with individually adapted intensity [21,22]. Work was done with progressive intensity, starting from easy to hard up to maximum values of 13 of the Borg scale (relatively hard effort). During the performance of the PTP, the face and the respiratory rate were monitored and a constant dialogue was maintained with the patient to monitor tolerance to the effort. As in Kraemer’s study, the large muscle group exercises were performed before the small muscle group exercises and the multijoint exercises before the single-joint exercises [23].

As for dexterity, by comparative assessment of the initial and final results obtained in the SWCWR the positive impact that the PTP has had on the ability of patients with MS in an incipient form of the disease is noted. As MS progresses, the ability to concentrate and fine movements are increasingly affected so that fine motor ability is reduced and PTP benefits are decreasing [25]. Moreover, regarding the impact of the PTP on finger dexterity, another aspect noted during our study, was that the benefits in the dominant hand are higher than in the nondominant hand. This is also confirmed by the results obtained in other specialized studies [24,25].

Upper limb dexterity was strongly associated with improved mood and a good ability to perform ADLs in particular. These results are consistent with other specialized studies. Bertoni et al. [284] concluded that loss of manual dexterity is associated with decreased independence in ADL and is likely due to a combination of tremor and sensory and strength disturbances. In the same vein, Cattaneo et al. [296] observed that impairment of manual dexterity was associated with restrictions in activities such as dressing, bathing, or cooking. The positive effects of RT on the dexterity of the upper limbs in patients with MS were also observed in the study by Ortiz-Rubio, who found clinical improvements regarding the precision of movements, the execution times, and the efficiency of certain functional tasks [27].

Even if in the SWCWR the cognitive performance improved significantly, the evolution of the cognitive performance was not significant between the two groups. Thus, we cannot state that the physical therapy caused the improvement.

Improving the mood with subsequent reduction in depression and cognitive impairment is also an important aspect of the PTP [28,29]. The PTP reduces stress and helps patients to be more relaxed, aspects that clearly improve the mood of MS patients [30]. As for the work technique of these exercises, it is very important for the physiotherapist to direct the exercises and to teach the patient to focus on the correctness of the exercises.

The regular performance of PTP determines the improvement in attention and memory as well as the increase in the speed of information processing in patients with MS. The more advanced the MS, the more obvious the cognitive impairment [31].

The PTP needs to be individualized and flexible and adjusted in terms of intensity, frequency, and the duration of the exercises, depending on each individual patient. It is recommended for the patient to be frequently assessed. This aspect has a double advantage: on the one hand, the therapy is updated and adjusted according to the patient’s condition and, on the other hand, the patient can perform self-assessment, which can represent a self-motivation [32].

OT addresses all the patient’s ADLs, aiming at keeping the patient engaged in social and independent self-care activities for as long as possible. There are studies in the literature that confirm the benefits of OT in which MS patients learn to manage their functional impairments and maintain the highest possible functional level, thus improving their quality of life [33,34].

There are clear benefits of a PTP in patients with MS, especially in those with mild to moderate disability, as shown by scores of 1.0–5.5 on the EDSS [35]. Available data suggest that an adapted PTP has positive effects in the fields of memory/learning, information processing, and attention/concentration [36]. Motl et al. claim that a PTP may be associated with reduced relapse rate and disease progression in MS [37]. In the study conducted by Campos and Toldrá it was noted that adapted OT activities have benefits in terms of symptoms, improved functioning, and therefore QoL in MS patients [38].

One of the most common symptoms of MS is depression and many recent studies provide explanations for its mechanism [39–41], as well as treatment possibilities [42,43]. There is a strong connection between performing the PTP at certain intensity and the patient’s state of mind. As the patient’s ability to perform the PTP declines, depression worsens and cognitive ability declines [44]. In patients with MS, the state of fatigue and the level of depression are significantly and directly influenced by the ability to perform physical activity [45].

In our study, the SWCWR improved both their mobility and leg function and cognitive performance. Similar results were noted in other studies that showed that exercise improved walking ability, depressive symptoms, fatigue, and several domains of cognitive function [46].

Learmonth et al. emphasize in their review the importance of physical exercise in the management of symptoms in patients with MS. They showed the benefits of PTP for numerous symptoms in MS and conclude that PT intervention should be prescribed early and performed with high frequency among those with MS [7].

The results of our study, although encouraging, are also limited by several methodological limitations. The study was conducted in a single center and the sample size was small. The number of patients with MS is low in Bihor County as the County Emergency Hospital, together with the Multiple Sclerosis Foundation in the city, ensures the rehabilitation of this condition. Ultimately, future studies should consider midterm follow-up assessments.

The results of our study reflect the advantages of combining DT with RT in MS therapy with the following benefits:

- functional improvement of the lower limbs;
- increased finger dexterity;
- amelioration of the mood and the ability to concentrate.

The indication for the PTP and OT is long term, practically whole life, mandatory, and at the patients’ home.

## Figures and Tables

**Table 1:** Initial characteristics of the groups included the study.

Characteristics	Group A (n = 39)	Group B (n = 38)	p
**Age** (**years**)	33.46 ± 4.506	33.13 ± 4.301	0.743
**Sex** (**%**) **male female**	33.3	31.6	0.869
	66.7	68.4	
**Environmental origin (%) urban rural**	71.8	68.4	0.746
	28.2	31.6	
**EDSS**	4.17 ± 1.47	4.15 ± 1.56	0.789

*Legend:* A—group with rehabilitation treatment, B—group without rehabilitation treatment, EDSS - Expanded Disability Status Scale, p values= statistical significance.

**Table 2:** Comparison of the initial values of studied parameters in the two groups (95% CI).

Studied Parameters	Group A	Group B	p	95% CI [lower/upper]
T25FW	7.605 ± 0.774	7.600 ± 0.761	0.976	−0.340/0.350
9HPT – dominant hand	19.803 ± 1.571	19.774 ± 1.553	0.936	−0.680/0.738
9HPT – nondominant hand	20.074 ± 1.639	20.013 ± 1.655	0.871	−0.686/0.738
PASAT	44.72 ± 5.605	44.18 ± 5.291	0.997	−2.514/0.504

*Legend:* group A—group with rehabilitation treatment, group B—group without rehabilitation treatment, p values = statistical significance, T25FW - Timed 25-Foot Walk, 9HPT - Nine-Hole Peg Test, PASAT - Paced Auditory Serial Addition Test.

**Table 3:** The evolution of values for studied parameters within groups and comparison of these variables between the groups (95% CI).

	Group A (n = 39)		Group B (n = 38)		Interaction	Effect size	Group A changes		Group B changes	
	Baseline (mean ± SD)	Post (mean ± SD)	Baseline (mean ± SD)	Post (mean ± SD)	p	p	95% CI Lower/Upper	p	95% CI Lower/Upper	p
T25FW test	7.605 ± 0.774	6.864 ± 0.761	7.600 ± 0.761	7.463 ± 0.863	**0.002** ^*^	0.122	0.648/ 0.834	**0.000** ^*^	0.029/0.245	0.014
9HPT – dominant hand	19.803 ± 1.571	17.474 ± 1.714	19.774 ± 1.553	19.639 ± 1.728	**0.000** ^*^	0.289	1.985/ 2.672	**0.000** ^*^	−0.085/0.353	0.222
9HPT – nondominant hand	20.074 ± 1.639	20.133 ± 0.290	20.013 ± 1.655	20.150 ± 0.295	0.868	0.000	−0.129/0.011	0.098	−0.201/−0.073	**0.000** ^*^
PASAT	44.72 ± 5.605	47.103 ± 1.144	44.18 ± 5.291	44.579 ± 0.866	0.084	0.039	−3.735/−0.034	**0.001** ^*^	−1.237/0.448	0.349
